# *ABCA3* deficiency from birth to adulthood presenting as paediatric interstitial lung disease

**DOI:** 10.1002/rcr2.633

**Published:** 2020-08-07

**Authors:** Jin‐Gun Cho, Devesh Thakkar, Peter Buchanan, Nicole Graf, John Wheatley

**Affiliations:** ^1^ Ludwig Engel Centre for Respiratory Research Westmead Institute of Medical Research Westmead NSW Australia; ^2^ Department of Respiratory and Sleep Medicine Westmead Hospital Westmead NSW Australia; ^3^ Westmead Clinical School, Faculty of Medicine and Health The University of Sydney Westmead NSW Australia; ^4^ Department of Respiratory Medicine Liverpool Hospital Liverpool NSW Australia; ^5^ Histopathology Department The Children's Hospital at Westmead, Sydney Children's Hospital Network Westmead NSW Australia; ^6^ The Children's Hospital Westmead Clinical School, Faculty of Medicine and Health The University of Sydney Westmead NSW Australia; ^7^ School of Medicine Western Sydney University Campbelltown NSW Australia

**Keywords:** *ABCA3* deficiency, interstitial lung disease, paediatric lung disease, surfactant dysfunction

## Abstract

Paediatric disorders of pulmonary surfactant may occur due to mutations involving surfactant proteins B and C, and ATP‐binding cassette subfamily A member 3 (*ABCA3*) genes. Recessive frameshift or nonsense *ABCA3* mutations are associated with respiratory failure and neonatal death but milder phenotypes of *ABCA3* deficiency due to missense, splice site, and insertion/deletions may result in survival beyond infancy. To date, only one case report describes the clinical course from birth to age 21 years and there are less than 10 adult cases. No guidelines exist for medical therapy due to the rarity of this condition. We describe the clinical course of a patient over 39 years and her younger brother who were both diagnosed at birth with an unspecified paediatric interstitial lung disease (ILD) and were eventually diagnosed with *ABCA3* mutation in their adulthood. Our report highlights the minimal progression of the *ABCA3*‐related ILD without long‐term medications, but the development of dyspnoea due to progressive pulmonary hypertension and airflow obstruction.

## Graphical abstract

We describe the clinical progress of siblings who initially presented with an undifferentiated paediatric interstitial lung disease at birth which was eventually diagnosed in adulthood as pulmonary surfactant dysfunction due to ATP‐binding cassette subfamily A member 3 (*ABCA3*) mutations. Clinical deterioration was due to progressive pulmonary hypertension and a moderate decline in forced expiratory volume in 1 sec (FEV_1_), rather than progression of the interstitial lung disease.

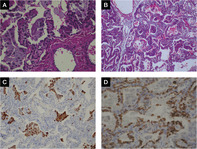

## Introduction

Pulmonary surfactant consists of a mixture of proteins and lipids which are produced by type II alveolar epithelial cells and are essential in reducing surface tension at the air–liquid interface within the lungs. Genetic disorders of surfactant production include surfactant protein B, surfactant protein C, and the ATP‐binding cassette subfamily A member 3 (*ABCA3*) [1]. *ABCA3* surfactant protein disorder is an autosomal recessive condition resulting in loss of function of the phospholipid transporter involved with pulmonary surfactant [2]. More specifically, *ABCA3* transports surfactant phospholipids into specialized secretory organelles known as lamellar bodies [3]. Although paediatric interstitial lung disease (ILD) due to mutations in the *ABCA3* gene has been increasingly recognized [4], there is very little information about this condition and its clinical course from birth to adulthood in milder forms of this disease [5].

## Case Report

A 25‐year‐old woman was referred for review of a long‐standing history of exertional dyspnoea since infancy.

She had a history of recurrent lower respiratory tract infections in her first year of life, associated with respiratory distress and diffuse interstitial changes. There was no family history of respiratory illness and both parents were healthy. On each admission, she was administered oxygen and antibiotics for presumed aspiration pneumonia. At five months, she was readmitted to a paediatric unit; multiple investigations were performed, including fibre‐optic bronchoscopy, immunoglobulins, sputum cultures, sweat electrolytes, and milk precipitins, which were all unremarkable. A radionuclide gastro‐oesophagram (milk scan) only revealed moderate‐to‐gross reflux in the prone position without evidence of pulmonary aspiration.

**Figure 1:**
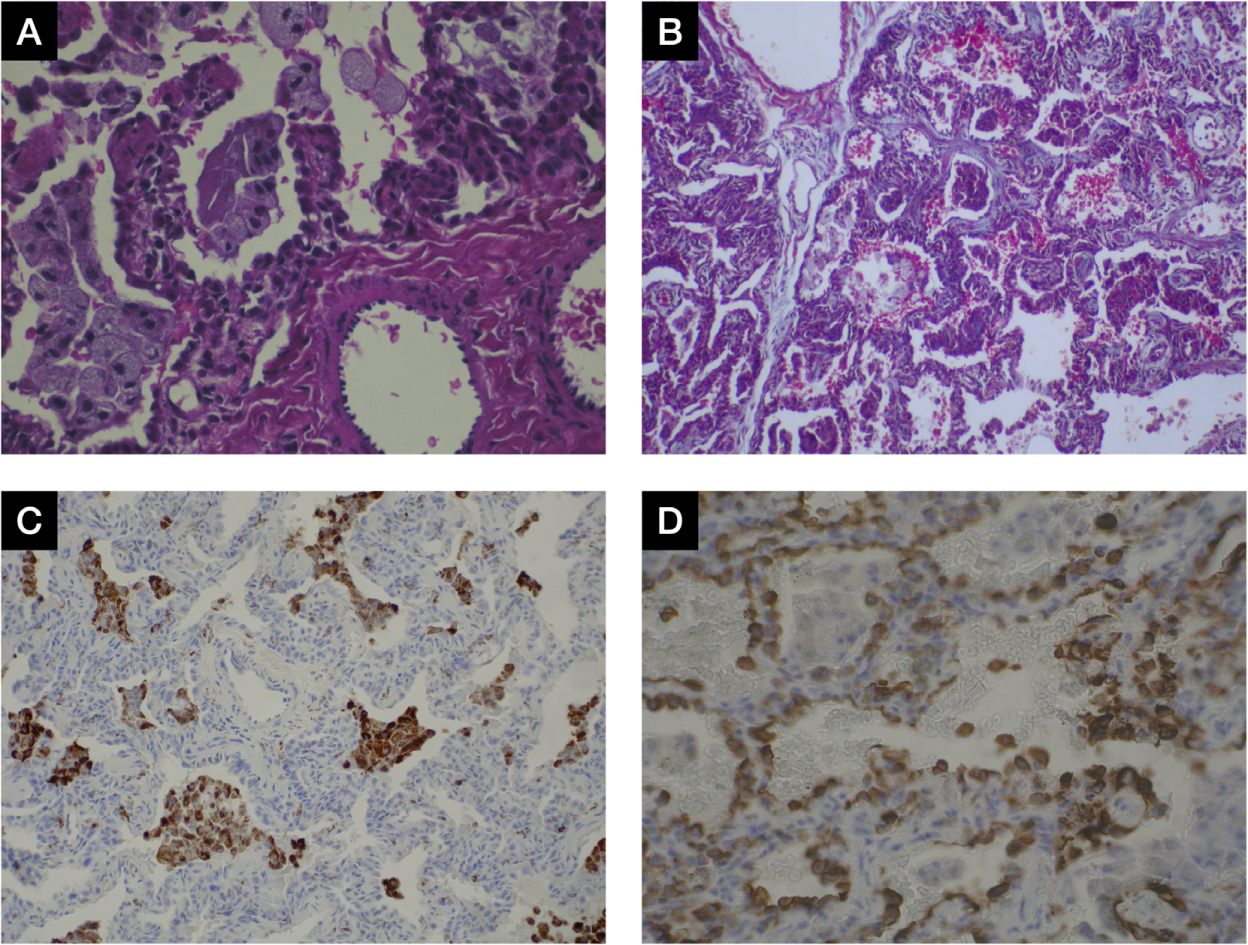
Open lung biopsy specimens in the older sister (age 11 months). (A) Haematoxylin and eosin (H&E)‐stained image (original magnification: 40×) showing pneumocyte hyperplasia with prominent intra‐alveolar macrophages, some with a xanthomatous appearance. A small amount of dense eosinophilic proteinaceous material is also seen within the alveolar space. There are no changes of pulmonary hypertension seen. (B) Masson's trichrome‐stained section (original magnification: 4×) showing thickening of the alveolar septa with patchy collagen deposition, and cellular aggregates within many of the alveoli resulting in a desquamative interstitial pneumonia (DIP)‐like pattern. (C) Immunohistochemistry for CD68 (original magnification: 10×) highlights prominent intra‐alveolar macrophages. (D) Immunohistochemistry for low molecular weight cytokeratin (original magnification: 40×) highlights the prominent enlarged pneumocytes, with occasional desquamated cells in the alveolar space.

An open lung biopsy was obtained at 11 months of age which demonstrated desquamated pneumocytes and a few foam cells (Fig. 1A) as well as fibrous thickening of alveolar septa and epithelialization of lining cells (Fig. 1B) with immunohistochemistry for CD68 highlighting prominent intra‐alveolar macrophages (Fig. 1C) and cytokeratin showing prominent enlarged pneumocytes (Fig. 1D) leading to a histopathological conclusion of pulmonary interstitial fibrosis of uncertain aetiology. She was treated with daily oral prednisolone from 11 months to four years of age at a starting dose of 2 mg/kg/day. When she was five years old, her younger brother was born with similar but milder clinical features and did not require hospitalization during his childhood. However, he was also treated with daily oral prednisone from five to seven years of age.

Her quality of life appeared near normal apart from some exercise limitation in her adolescent years. At age 25 years, she was seen by an adult respiratory physician having had a diagnosis of a paediatric ILD. Physical examination revealed finger clubbing and bilateral lower zone fine inspiratory crackles on auscultation. An arterial blood gas sample on room air demonstrated moderate hypoxaemia with partial pressure of oxygen (PaO_2_) 61 mmHg, partial pressure of carbon dioxide (PaCO_2_) 33 mmHg, and arterial oxygen saturation (SaO_2_) 95%.

In view of her unusual history and clinical features, a surfactant protein deficiency disorder was suspected. DNA from both siblings was extracted from whole blood and sent to the Department of Pediatrics Research Laboratory at Johns Hopkins University School of Medicine which revealed compound heterozygosity for a known *ABCA3* mutation (c.3609_11delCTT; F1203del) and a second, previously unknown, missense mutation in *ABCA3* exon 5 (c.127 C>T; p.R43C) [4]. Genetic testing of parents showed that her mother was heterozygous for the F1203del mutation and her father was heterozygous for the p.R43C mutation.

**Figure 2:**
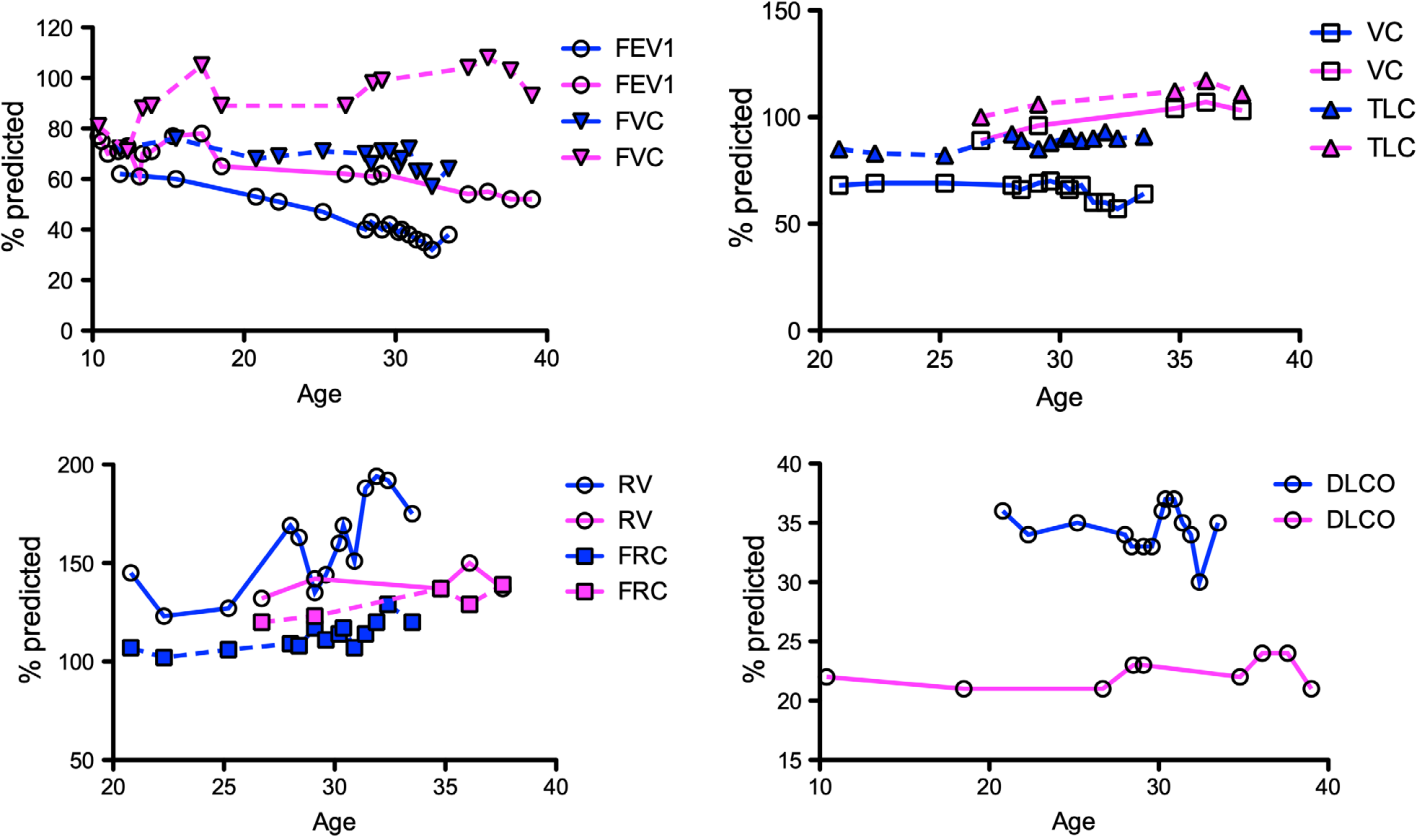
Serial lung function tests for the sister (purple) and brother (blue) from age 10.5 to 39 years. Forced expiratory volume in 1 sec (FEV_1_) gradually decreased in both over time. Total lung capacity (TLC) and vital capacity (VC) as measured by plethysmography remained stable over time. There was a trend towards increasing residual volume (RV) and functional residual capacity (FRC) in both as airflow obstruction increased in adulthood. Diffusing capacity was severely reduced and remained unchanged.

**Figure 3:**
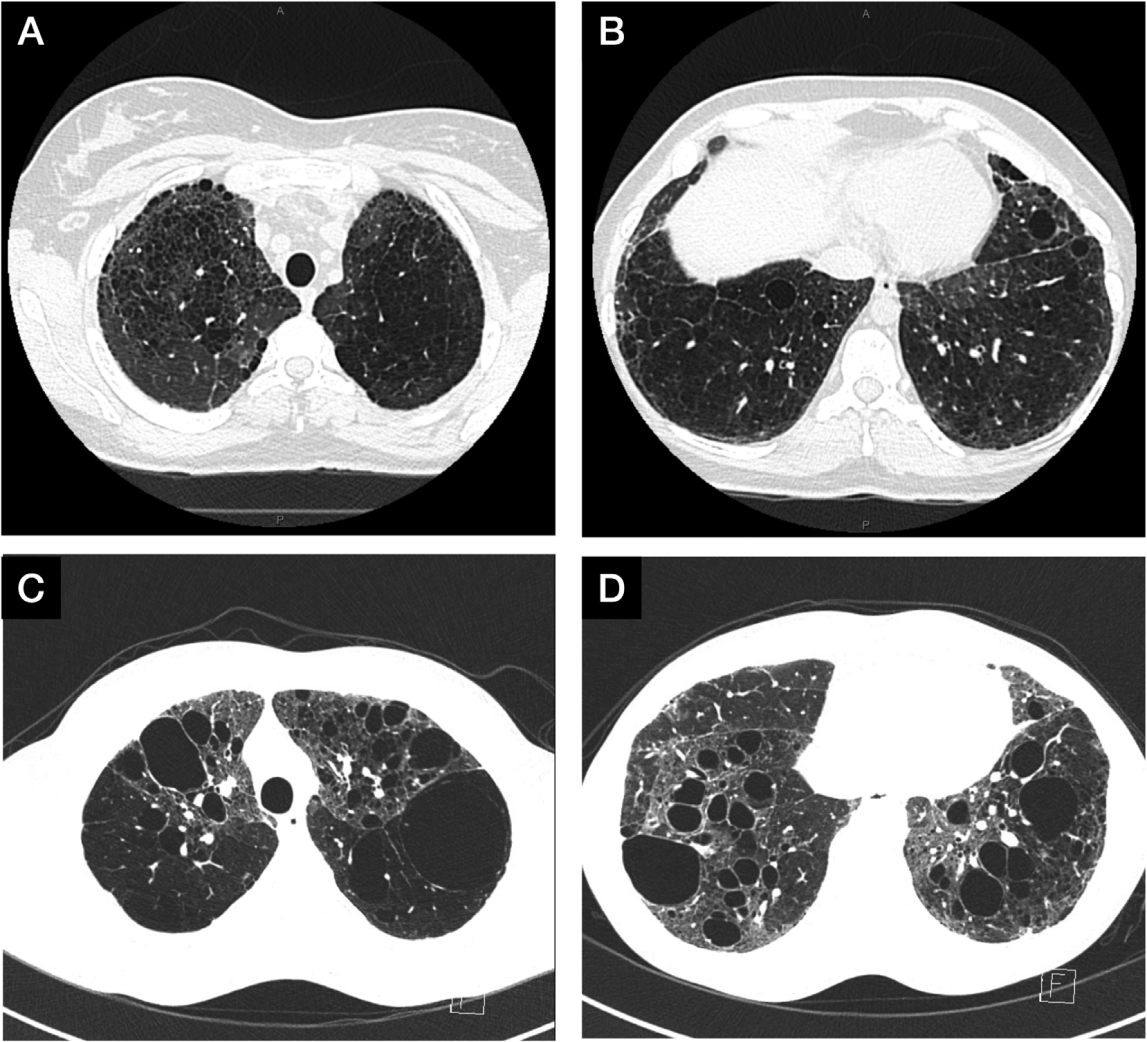
High‐resolution computed tomography (HRCT) scan of the chest for the sister (A and B) and the brother (C and D). (A) HRCT of the chest at age 28 years demonstrating extensive cystic change in the upper lobes including subpleural regions. There is a small area of normal appearing lung in the posterior segment of the right upper lobe. (B) There is extensive vascular attenuation in the lower lobes with multiple well‐defined cysts up to 20 mm in size at the bases. (C and D) HRCT of the chest at age 30.5 years demonstrating extensive thin walled cysts measuring from a few millimetres to 60 mm. There are also areas of reticulation and parenchymal distortion. The cysts are larger and more prominent in the brother than the sister.

Her serial pulmonary function tests demonstrated an obstructive pattern with forced expiratory volume in 1 sec (FEV_1_) gradually decreasing from 78% predicted at age 10.5 years to 52% predicted at age 39 years, but forced vital capacity (FVC) remained within the normal range at 93% predicted at age 39 years. Diffusing capacity was severely reduced below 25% predicted from age 10.5 years and did not change over time (Fig. 2). Plethysmographic lung volumes did not reveal any lung restriction. A high‐resolution chest computed tomography (HRCT) scan at age 28 years showed severe diffuse lung disease with extensive distribution of cystic change throughout the lungs (Fig. 3A). In the lower lobes there was extensive vascular attenuation, and at the bases were multiple well‐defined cysts up to 20 mm size (Fig. 3B). Comparison to an HRCT chest three years prior showed no interval change. Transthoracic echocardiography at age 27 years showed an elevated pulmonary pressure at 55 mmHg with mild right ventricular and right atrial enlargement but normal contractility.

Her brother had a similar obstructive pattern on serial lung function testing but had developed more airflow obstruction over time in conjunction with a higher residual volume and diffusing capacity (Fig. 2). His HRCT chest at age 30.5 years also demonstrated extensive thin walled cysts measuring from a few millimetres to 60 mm. There were areas of reticulation and parenchymal distortion consistent with fibrosis with air trapping on expiratory series (Fig. 3C, D).

With genetic counselling and in view of the autosomal recessive nature of *ABCA3* deficiency, the patient sought pregnancy at age 27 years, despite her cardiorespiratory disease. She was managed in a high‐risk obstetrics unit from 30 weeks gestation, and given inhaled iloprost two weeks pre‐partum and immediately post‐partum. Elective lower segment caesarean section delivered a healthy male baby without any lung disease.

She was managed conservatively with supplemental oxygen after she developed resting hypoxaemia from age 27 years. No specific drug therapy for *ABCA3* deficiency was trialled and pulmonary hypertension medications were unable to be continued due to patient‐perceived side effects. She developed right heart failure at age 39 years and was subsequently referred for consideration of a lung transplant. Her brother was also diagnosed with pulmonary hypertension and had been managed in a pulmonary hypertension clinic since age 28 years. He has remained on room air and is maintained on tadalafil and macitentan as well as fluticasone, umeclidinium, and vilanterol inhaler.

## Discussion

*ABCA3* mutations in adults with ILD are extremely rare with a lung registry study identifying only three adult cases [6], and a recent literature review finding nine adult cases with only one of the cases being diagnosed at birth and followed up through adulthood [5]. Our report demonstrates the interesting observation of *ABCA3* mutations in siblings resulting in cystic lung disease, fibrosis, and airflow obstruction with a gradual decrease in FEV_1_ over 30 years, but otherwise essentially stable pulmonary disease and lung function over 39 years into adolescence and adulthood. Her eventual declining clinical status was due to progression of pulmonary hypertension rather than her ILD. This observation is consistent with findings in children where mean lung function was low but tended to remain unchanged [7]. Although both siblings only tested positive for one known mutation (F1203del), the other abnormality consisted of a missense mutation in exon 5 (p.R43C, a substitution of cysteine for arginine) which was not recognized in *ABCA3* deficiency at the time of testing of these siblings [4]. However, compound heterozygotes with mutations in the same codon (p.R43L and p.R43H) have been previously noted, and two other unrelated *ABCA3* deficiency cases involving p.R43C missense mutations have been subsequently documented [4], making it likely that the p.R43C mutation was responsible for the clinical phenotype. Even with the same genetic mutations within the siblings, there is some clinical variability with the sister having less airflow obstruction but more severe pulmonary hypertension, and the brother having greater airflow obstruction, more extensive cysts, and gas trapping.

Although recessive frameshift or nonsense *ABCA3* mutations are associated with respiratory failure and neonatal death [2, 4], milder forms of *ABCA3* deficiency exist which may be associated with survival beyond infancy [8]; these milder phenotype may occur with missense, splice site, and insertion/deletion *ABCA3* mutations [4]. The presence of multiple parenchymal cysts may be a feature of this condition [6] and was previously noted in five out of nine children in a case series [7] and also in adults [5]. Lung histopathology may include a variety of presentations including pulmonary alveolar proteinosis, desquamative interstitial pneumonitis, and non‐specific interstitial pneumonitis, but the characteristic feature of *ABCA3* mutations is dense formation of lamellar bodies on electron microscopy [7]. Definitive diagnosis is via genetic testing for *ABCA3* mutations, although a probable diagnosis of a genetic basis may be made on lung biopsies with immunohistochemistry and electron microscopy [1].

There are currently no guidelines for drug therapy to treat patients with ILD due to *ABCA3* mutations, although prednisone, azathioprine, azithromycin, whole lung lavage, and prednisone with hydroxychloroquine have been trialled [5]. It is likely that lung transplantation is the only “curative” procedure available; however, transplant data are limited to infants and children with only one adult case who underwent lung transplantation at age 21 years [5]. Outcomes from childhood transplantation for genetic disorders of surfactant metabolism including *ABCA3* deficiency remain poor due to growth impairment, bronchiolitis obliterans, and other transplant‐associated morbidities [9].

In conclusion, an *ABCA3* mutation may be considered in an adult if there are findings of diffuse pulmonary cysts and pulmonary fibrosis with a neonatal or childhood history of respiratory distress. Airflow obstruction and pulmonary hypertension may progress in adulthood, contributing to hypoxaemia and right heart failure. If a rare surfactant protein disorder is suspected, genetic testing and advice from a specialized unit are recommended.

### Disclosure Statement

Appropriate written informed consent was obtained for publication of this case report and accompanying images.
